# Supplementary material for: The cross-sectional association between state-level public health funding per capita and physical health among adults in the United States
Source: PLoS One. 2025 Apr 1;20(4):e0320920. doi: 10.1371/journal.pone.0320920 (PMC11960902; doi:10.1371/journal.pone.0320920)
Supplement: Attachment — Submitted filename: PONE-D-24-42416_R1.pdf [file pone.0320920.s002.pdf]

Abstract

Objectives: This study examined the association between state-level public health funding per capita and the odds of poor physical health.

Study Design: Cross-sectional

Methods: Data from the 2018 Behavioral Risk Factor Surveillance System (BRFSS) were used. Participants' self-reported physical health was reported using the CDC Healthy Days Core Module. State-level public health funding per capita was obtained from the State Health Access Data Assistance Center website. Multilevel logistic regression was used to adjust for self-reported individual-level characteristics and area-level characteristics from the 2018 American Community Survey. We also tested whether household income or education attainment moderated any observed associations

Results: A one SD increase in state-level public health funding per capita was not associated with the odds ≥ 14 days of poor physical health (OR= 0.95, 95% CI: 0.89, 1.04). However, heterogeneity across household income was observed. Greater public health funding per capita was associated with lower predicted probabilities of reporting ≥ 14 days of poor physical health among respondents from low household income backgrounds (< $35,000 USD) compared to participants with high household incomes (>$75,000 USD). No associations were observed among those with moderate ($35,000 – $70,000 USD) household incomes. A similar finding was observed among participants with less than high school education when compared to participants with post-secondary education.

Conclusion: Greater state-level public health funding per capita appears to have a protective association against reporting ≥ 14 days of poor physical health in individuals with lower

household incomes and may be helpful in reducing health inequities. Future research is needed to determine whether this association is causal.

Keywords: Inequities, social determinants, perceived health,

Introduction

Health related quality of life (HRQoL) is the perceptions of one's health [1]. Surveillance estimates appear mixed regarding trends in HRQoL since the millennium in the United States. While some studies have found stable or increasing trends in HRQoL [2, 3], others have found decreases [4]. HRQoL comprises of mental and physical components. Physical HRQoL is a stronger predictor of overall mortality, even after accounting for age, gender, and various health conditions [5]. In addition to its association with mortality, obtaining information about individual perspectives of their health has been deemed important for the allocation of resources and quality improvement in the health care system [6] and understanding the burden of living with chronic or infectious diseases [7-10].

Researchers have developed several models to investigate the determinants of HRQoL [11]. The most commonly used include those by Wilson & Cleary [12], Word Health Organization [13], and Ferrans and Zerwic [14]. Among these, the Ferrans, Zerwic (14) model has been recommended because it incorporates both individual and environmental (i.e., anything outside the individual) factors [11]. In their systematic review, Duangchan and Matthews (15) synthesized the literature that employed Ferrans, Zerwic (14) conceptual model of HRQoL and found individual level factors were the primary focus of the literature, with characteristics such as age, gender, and race being associated with HRQoL. Whereas others have found area level characteristics such as racial/ethnic concentrations to be associated with self reported health [16], and socioeconomic deprivation, an index based on age, education, housing conditions, housing expenditure, and unemployment to be associated with poor physical HRQoL [17].

a positive relationship between socioeconomic status and health.

The socioeconomic gradient in health suggests increasing levels of socioeconomic status will correspond to increasing health improvements [18]. It is possible that differences in HRQoL also follow a socioeconomic gradient. Evidence from systematic reviews of longitudinal studies have found some support regarding income and self-rated health, where greater income is associated with better self-rated health [19, 20]. Self-rated general health is closely related to perceptions of physical health. Therefore, it is reasonable to extend the results from these reviews to self-rated physical health [21]. As a result, identifying factors that can reduce health inequities, and by extension physical health are important.

likely that HRQoL

shows

line 76 and onwards, delete the green highlighted sections.

, which should improve

Public health aims to promote population health and reduce health inequities making it an upstream environmental factor that could impact HRQoL and reduce any socioeconomic disparities that may exist [14, 22]. To do this, public health units need adequate funding to retain staff and maintain programs and services [23]. More public health spending has been associated with various positive outcomes such as reduced infant-, cardiovascular-, diabetes-, and cancer-mortality [24] and rates of sexually transmitted diseases [25]. However, less is known about the association between public health funding and HRQoL. Therefore, the focus of this research was to examine the association between state-level public health funding and reporting poor physical health using data from the 2018 Behavioral Risk Factor Surveillance System (BRFSS). A secondary aim was to determine whether the association between public health spending and poor physical health was modified by individual household income or educational attainment

improve health and quality of life

Higher levels of

was found to be associated

of

of the individuals.

Methods

The BRFSS is an ongoing federal-state partnership surveillance program that collects data on health behaviours, chronic health conditions, and use of preventive services via telephone

surveys [26]. Participants consist of non-institutionalized individuals aged 18 years and above residing in the United States. Although the BRFSS is not population representative the use of survey weights helps make the data more representative to the non-institutionalized adult population. The BRFSS currently operates in all 50 states, as well as the district of Columbia [27]. For the current study, individual data from the 2018 BRFSS was linked with state level data from the State Health Access Data Assistance Center (SHADAC) and the 2018 America Community Survey. Informed written or verbal consent was not obtained by the study team as this was an analysis of publicly available secondary data (https://www.cdc.gov/brfss/annual_data/annual_2018.html). This study received approval from the Health Research Ethics Board at the University of Alberta (Pro00134036).

Main Exposure of Interest

For each US state and the District of Columbia, data on per capita state public health funding for the year 2018 was obtained from the State Health Access Data Assistance Center (SHADAC) website [28]. The public health funding variable summarizes funds allocated to infectious disease control, chronic disease prevention, injury prevention, environmental public health, maternal, child, and family health, and access to a linkage with clinical care. The Trust for America's Health created this variable based on funding information made publicly available from each state [29]. Per capita state public health funding was then z-transformed to help with interpretation.

Main Outcome of Interest –Physical Health

Self-reported days of poor physical health was collected via one item from the Center for Disease Control and Prevention's Healthy Days Measures [7]. Specifically, participants were

the following question to obtain data on

asked to report on their self-rated physical health: “Now thinking about your physical health, which includes physical illness and injury, for how many days during the past 30 days was your physical health not good?” Responses could select between 0 to 30 days, none, don’t know/not sure. This measure has shown good construct validity with self-perceived health status [30]. For the analysis, self-report of poor physical health in the last 30 days was binary coded as < 14 days of poor physical health and ≥14 days of poor physical health. This cutoff of 14 days or more has been previously used with the BRFSS healthy days module [6, 31, 32]

was previously used by

Covariates

Individual-level characteristics were included based on their strong associations with physical health. Age, sex assigned at birth (male; female); from which we interpret as gender given the focus on the manuscript is on social influences rather than biological mechanisms; racial identity, marital status, educational attainment, and annual household income. For the current study, racial identity (considered as another social construction) was coded into 6 categories: Non-Hispanic White; Non-Hispanic Black or African American; American Indian or Alaska Native/Pacific Islander; Hispanic; Asian; Other). Marital status was coded as single (divorced, widowed, separated, never married) or coupled (married, a member of an unmarried couple). Educational attainment was coded into three categories: less than high school, high school graduate, and college graduate. Annual household income was coded as low (less than $35,000), medium ($35,000 to $75,000), and high (greater than $75,000). Area-level confounders included proportion of residents who are Black, population, median household income, proportion living in poverty, and were derived from the 2018 American Community Survey (ACS) which is publicly available [33]. Additionally, we adjusted for the type of public health structure based on previously established classifications [34]

Statistical Analysis

All analyses were performed in HLM (version 7.20) and used the final weights assigned to individuals. Multilevel logistic regression analyses were performed to account for the clustered structure of the data (individuals within states) while adjusting for individual- and area-level confounders and survey weights. First, a null model was performed to obtain the overall predicted probability of ≥ 14 days of poor physical health and 95% plausible value range. Next, an unadjusted model with only public health funding per capita was performed followed by an adjusted model with individual and area-level confounders included. Finally, cross-level interaction terms for household income*public health funding per capita were included in the fully adjusted model. This was repeated for educational attainment in a separate analysis. Statistical significance was determined a priori ($p<0.05$).

Results

There were 348,221 participants from 50 states and the District of Columbia included in the analysis. Descriptive statistics for individual- and area-level characteristics are presented in Table 1. Just over half (53.4%) of the weighted sample was aged 45 years or older, were females (50.0%) or were partnered (56.8%). Just under two-thirds of the weighted sample were Non-Hispanic White (64.6%), had some college or post-secondary education (61.3%), or had medium to high incomes (64.5%). At the area-level, the median population size was 4,461,153 (IQR = 5,773,333), with 11.8 % (SD = 10.7), and 12.9% (2.8), of the population on average being Black, or living in poverty, respectively. The average state public health funding per capita was $42.0 USD (SD = $30.7 USD) per capita, and the average median state income was $59,955 USD (SD =10,585 $ USD).

Table 1 Descriptive characteristics of the 2018 BRFSS participants (n = 328, 221) and area level factors (n = 51) included in the analysis

**Individual Characteristics**	Sample Frequency (Weighted Percent)	
Age		
18-24	18, 459 (11.02%)	
25-44	83, 868 (35.58%)	
45-64	130, 491 (34.08%)	
65+	115, 403 (19.32%)	
Gender		
Male	163, 178 (49.96%)	
Female	185, 043 (50.04%)	
Race		
Non-Hispanic White	269, 101 (64.59%)	
Non-Hispanic Black or African American	28, 687 (11.58%)	
Asian	7, 752 (5.07%)	
American Indian or Alaska Native/Pacific Islander	6, 497 (1.00%)	
Hispanic	25, 479 (15.75%)	
Other	10, 705 (2.00%)	
Marital Status		
Partnered	196, 373 (56.81%)	
Single	151, 848 (43.19%)	
Household income		
Low	122, 191 (30.12%)	
Medium	104, 848 (28.18%)	
High	121, 136 (36.28%)	
Education		
Less than high school	22,718 (11.84%)	
High school	90, 887 (26.87%)	
Some college	96, 858 (31.62%)	
Post-secondary	137, 758 (29.67%)	
**Area-level characteristics**	Mean (SD)	Range
Population	6,405,637 (7,327,258)	57,7601 – 39,500,000
Proportion Black (%)	11.83 (10.72)	0.6 – 46.4
Median income ($USD)	62,034.65 (10,584.69)	44097 – 85203
Proportion living in poverty (%)	12.91 (2.84)	7.6 – 19.7
Public Health Funding ($USD)	42.04 (30.68)	7.07 – 135.32

Note: IQR = Interquartile range

The overall predicted probability for ≥ 14 days of poor physical health was 12.5% (95% plausible value range: 9.6%, 15.6%; Table 2). In the unadjusted model, a one SD increase in public health funding per capita was not associated with the likelihood of reporting poor physical health ≥ 14 days (OR= 0.97, 95% CI: 0.91, 1.04). Similar results were observed after adjusting for individual and area-level characteristics (OR =0.96, 95% CI:0.90, 1.01; Table 2).

The household income*public health funding per capita interaction was significant (p=0.007) for lower income households and the results are displayed in Fig 1. The results suggest that increased public health funding per capita is associated with a lower probability of reporting poor physical health > 14 days among individuals in the lowest income group and the slope is different compared to individuals from high income households. Educational attainment revealed a similar pattern (Fig 2). Participants with less than high school education had lower predicted probabilities of poor physical health compared to those with post-secondary education (Table 2).

Table 2 Multilevel multiple logistic regression modelling the association between individual and area-level characteristics with ≥14 days of poor physical health using data from the 2018 BRFSS, SHADAC, and 2018 ACS among all states

Fixed Effect	OR (95% CI)	AOR (95% CI)[a]	AOR (95% CI)[b]	AOR (95% CI)[c]
**Area-level characteristics**				
z-PH Funding	0.97 (0.91, 1.04)	0.96 (0.90, 1.01)	1.01 (0.93, 1.09)	0.98 (0.91, 1.05)
Population		0.98 (0.94, 1.02)	0.98 (0.94, 1.02)	0.98 (0.94, 1.02)
Proportion Black		**0.94 (0.89, 1.00)**	**0.94 (0.89, 1.00)**	**0.94 (0.89, 1.00)**
Median income		**1.15 (1.04, 1.27)**	**1.15 (1.03, 1.27)**	**1.15 (1.03, 1.27)**
Proportion living in poverty		**1.18 (1.07, 1.30)**	**1.18 (1.07, 1.30)**	**1.18 (1.07, 1.30)**
PH Governance Typology				
Central		0.97 (0.88,1.07)	0.97 (0.88, 1.07)	0.97 (0.88, 1.07)
Mixed/Shared		1.08 (0.98, 1.19)	1.08 (0.98, 1.19)	1.08 (0.98, 1.19)
Decentralized		Reference	Reference	Reference
**Individual-level characteristics**				
Age				

18-24	**0.19 (0.16, 0.22)**	**0.19 (0.16, 0.22)**	**0.19 (0.16, 0.22)**
25-44	**0.47 (0.44, 0.50)**	**0.47 (0.44, 0.50)**	**0.47 (0.44, 0.50)**
45-64	**1.12 (1.06, 1.18)**	**1.12 (1.06, 1.19)**	**1.12 (1.06, 1.19)**
65+	Reference	Reference	Reference
Gender			
Male	Reference	Reference	Reference
Female	1.04 (0.99, 1.09)	1.04 (0.99, 1.09)	1.04 (0.99, 1.09)
Race			
Non-Hispanic White	Reference	Reference	Reference
Non-Hispanic Black or African American	**0.79 (0.73, 0.86)**	**0.79 (0.73, 0.86)**	**0.79 (0.73, 0.86)**
Asian	**0.59 (0.46, 0.75)**	**0.59 (0.46, 0.75)**	**0.58 (0.46, 0.75)**
American Indian or Alaska Native/Pacific Islander	**1.35 (1.16, 1.59)**	**1.35 (1.16, 1.59)**	**1.36 (1.16, 1.59)**
Hispanic	**0.68 (0.61, 0.75)**	**0.68 (0.61, 0.75)**	**0.68 (0.61, 0.75)**
Other	**1.25 (1.11, 1.41)**	**1.25 (1.11, 1.41)**	**1.25 (1.11, 1.41)**
Marital Status			
Partnered	Reference	Reference	Reference
Single	**1.10 (1.04, 1.16)**	**1.10 (1.04, 1.16)**	**1.10 (1.04, 1.16)**
Household income			
Low	**3.73 (3.45, 4.03)**	**3.69 (3.41, 3.98)**	**3.74 (3.46, 4.04)**
Low*z-PH Funding		**0.92 (0.86, 0.99)**	
Medium	**1.72 (1.59,1.86)**	**1.72 (1.60, 1.86)**	**1.73 (1.60, 1.86)**
Medium*z-PH Funding		0.98 (0.90, 1.06)	
High	Reference	Reference	Reference
Education			
Less than high school	**2.11 (1.92, 2.31)**	**2.11 (1.93, 2.32)**	**2.06 (1.88, 2.26)**
Less than high school*z-PH Funding			**0.89 (0.81, 0.98)**
High school	**1.59 (1.48, 1.70)**	**1.59 (1.49, 1.70)**	**1.59 (1.48, 1.70)**
High school*z-PH Funding			0.99 (0.92, 1.06)
Some college	**1.51 (1.41, 1.61)**	**1.51 (1.41, 1.61)**	**1.51 (1.41, 1.61)**
Some college*z-PH Funding			1.01 (0.94, 1.08)
Post-secondary	Ref	Ref	Ref

Note: Bolded values are significant $p<0.05$. OR = Odd's Ratio, 95% CI = 95% confidence intervals. [a] Adjusted for individual and area level characteristics. [b] Adjusted model including the interaction term (z-Public Health Funding * household income). [c] Adjusted model including the interaction term (z-Public Health Funding * educational attainment). Abbreviations: PH = Public health

Fig 1. Predicted probabilities of poor physical health associated with public health funding per capita by household income

Note: This figure is displaying the predicted probabilities of reporting ≥14 days of poor physical health associated with public health funding per capita by household income. Figure 2

Fig 2. Predicted probabilities of poor physical health associated with public health funding per capita by education attainment

Note: This figure is displaying the predicted probabilities of reporting ≥14 days of poor physical health associated with public health funding per capita by educational attainment.

Discussion

Our study examined whether public health funding per capita was associated with physical health and explored any potential heterogeneity by household income. Based on our cross-sectional results, public health funding per capita was not associated with poor physical health, though heterogeneity was observed by household income and educational attainment. Specifically, more public health funding per capita was associated with a lower likelihood of reporting physically unhealthy days in participants from lowest income groups and lowest education groups, whereas the likelihood of reporting physically unhealthy days among middle- and higher-income, and higher education participants remained relatively stable and not associated across quartiles of public health funding per capita. These findings are supportive of increasing funding for public health in the United States given their goal of promoting health in low socioeconomic status populations thereby reducing health inequities.

The non-significant association for public health funding and physical health may be explained by the cross-sectional nature of the data and the inability to detect any potential lag effects that public health funding may have on physical health [35]. The lack of association could also be explained by the concept of vertical equity, where states with greater needs, in this case worse physical health, received higher funding [36]. Given the significant interactions between household income and educational attainment withpublic health funding that we observed, it is possible that states with greater funding were equipped with programming, staffing, and surveillance or monitoring that enabled them address inequities in physical health by targeting lower income and lower education households [23]. Either way, it is difficult to understand the mechanism through which public health funding impacts health behaviours and outcomes without further assessment of intermediary factors such as quality, effectiveness, or efficiency [37]. For example, prior research has found some evidence that increased state and federal funding was associated ($p<0.10$) with less efficiency by local health departments, however, greater efficiency was associated ($p<0.05$) with a greater proportion of programs being produced internally by the health departments [38]. As a result, future research will benefit from assessing why and how public health unit funding per capita is associated with decreased odds in reporting poor physical health in lower income groups.

Our results support the vulnerable population approach described by Frohlich and Potvin (39). For example, our findings regarding the entire sample demonstrated a null association between public health unit funding and poor physical health. Had interactions never been performed, we would have missed a key finding that public health unit funding per capita has protective associated with poor physical health for low-income and low-education participants (i.e., a vulnerable population). It is unclear whether our findings are consistent with the literature

as most other studies looking at public health funding and population health did not examine whether associations were heterogenous by household income or education [35]. The lack of association between public health funding and poor physical health among higher income and more educated participants may be explained by the fundamental causes articulated by Link and Phelan (40), where wealthier individuals are more likely to be able to obtain resources (i.e., knowledge, money, power, prestige, social connections) in order to benefit their health and receive diminishing returns from investment in public health, whereas people with lower incomes or lower educations do not have these resources and may rely more on public services.

Strengths of this study included the representative sample from which the BRFSS collects data on as well as the previously validated measures of physical health [7]. However, there are also some limitations. For example, this study was cross-sectional making it impossible to make causal inferences (e.g., state-level public health funding per capita is causally associated with health among people with low household income). This also reduced our ability to examine any potential lag effects that might be present between public health funding and individual physical health. Another limitation is the general nature of public health funding. Given public health has multiple standards to deliver on, it is difficult to know whether funding allocated to one area (e.g., disease prevention) versus another (e.g., health promotion) may improve physical health [23, 28]. Future research may benefit from using more nuanced data on public health funding. Finally, while we did control for state-level public health structure we did not control for other relevant constructs resembling the organizational capacity (e.g., organizational culture, human resources) or study any intermediary factors (e.g., quality, efficiency) that may lie between public health funding and physical health [37].

Conclusion

Based on the findings from this study it appears that state-level public health funding per capita was associated with a decreased odds in reporting poor physical health among participants with lower household incomes and less than high school education. This is an important finding for public health as it suggests that more public health funding at the state level could potentially reduce health inequities as indicated by the social gradient [18]. Future research may benefit from using longitudinal study designs to assess potential lag effects. Further, using more specific data (e.g., fund allocation) and assessment of intermediary factors (e.g., quality, efficiency) will help clarify the association between state-level public health funding and health outcomes.

Acknowledgements

None

References

1. Karimi M, Brazier J. Health, health-related quality of life, and quality of life: What is the difference? PharmacoEconomics. 2016;34(7):645-9. doi: 10.1007/s40273-016-0389-9.

2. Stewart ST, Cutler DM, Rosen AB. Comparison of trends in US health-related quality of life over the 2000s using the SF-6D, HALex, EQ-5D, and EQ-5D Visual Analog Scale versus a broader set of symptoms and impairments. Medical Care. 2014;52(12):1010-6. PubMed PMID: edsjsr.26417858.

3. Greaney ML, Cohen SA, Blissmer BJ, Earp JE, Xu F. Age-specific trends in health-related quality of life among US adults: Findings from National Health and Nutrition Examination Survey, 2001–2016. Quality of Life Research. 2019;28:3249-57.

4. Olfson M, Wall M, Liu S-M, Schoenbaum M, Blanco C. Declining health-related quality of life in the U.S. American Journal of Preventive Medicine. 2018;54(3):325-33. doi: https://doi.org/10.1016/j.amepre.2017.11.012.

5. Reinwarth AC, Wicke FS, Hettich N, Ernst M, Otten D, Brähler E, et al. Self-rated physical health predicts mortality in aging persons beyond objective health risks. Scientific Reports. 2023;13(1):19531.

6. Slabaugh SL, Shah M, Zack M, Happe L, Cordier T, Havens E, et al. Leveraging health-related quality of life in population health management: The case for healthy days. Population Health Management. 2017;20(1):13-22. doi: 10.1089/pop.2015.0162. PubMed PMID: 27031869.

7. Moriarty DG, Zack MM, Kobau R. The Centers for Disease Control and Prevention's Healthy Days Measures - population tracking of perceived physical and mental health over time. Health and Quality of Life Outcomes. 2003;1:37. Epub 20030902. doi: 10.1186/1477-7525-1-37.

8. Ansah JP, Chiu CT. Projecting the chronic disease burden among the adult population in the United States using a multi-state population model. Frontiers in Public Health. 2022;10:1082183. Epub 20230113. doi: 10.3389/fpubh.2022.1082183.

9. Hughes J, Jelsma J, Maclean E, Darder M, Tinise X. The health-related quality of life of people living with HIV/AIDS. Disability and Rehabilitation. 2004;26(6):371-6. doi: 10.1080/09638280410001662932.

10. Lofstrom E, Kunkel S, Kotz A, Lingman M, Unden J, Nygren JM. Health-related quality of life and long-term symptoms among patients with non-severe covid-19-a prospective cohort study. Infectious Diseases. 2023. doi: 10.1080/23744235.2023.2175904.

11. Bakas T, McLennon SM, Carpenter JS, Buelow JM, Otte JL, Hanna KM, et al. Systematic review of health-related quality of life models. Health and Quality of Life Outcomes. 2012;10(1):134. doi: 10.1186/1477-7525-10-134.

12. Wilson IB, Cleary PD. Linking clinical variables with health-related quality of life. A conceptual model of patient outcomes. JAMA. 1995;273(1):59-65. PubMed PMID: 7996652.

13. World Health Organization. International classification of impairments, disabilities, and handicaps: a manual of classification relating to the consequences of disease, published in accordance with resolution WHA29.35 of the Twenty-ninth World Health Assembly, May 1976: World Health Organization; 1980.

14. Ferrans CE, Zerwic JJ, Wilbur JE, Larson JL. Conceptual model of health-related quality of life. Journal of Nursing Scholarship. 2005;37(4):336-42. doi: https://doi.org/10.1111/j.1547-5069.2005.00058.x.

15. Duangchan C, Matthews AK. Application of Ferrans et al.'s conceptual model of health-related quality of life: A systematic review. Research in Nursing & Health. 2021;44(3):490-512. doi: https://doi.org/10.1002/nur.22120.

16. White K, Borrell LN. Racial/Ethnic Neighborhood concentration and self-reported health in New York City. Ethnicity & Disease. 2006;16(4):900-8.

17. Rocha V, Ribeiro AI, Severo M, Barros H, Fraga S. Neighbourhood socioeconomic deprivation and health-related quality of life: A multilevel analysis. PLOS ONE. 2017;12(12):e0188736. doi: 10.1371/journal.pone.0188736.

18. Marmot MG. Understanding social inequalities in health. Perspectives in Biology and Medicine. 2003;46(3):S9-S23.

19. Reche E, Koenig H-H, Hajek A. Income, self-rated health, and morbidity: A systematic review of longitudinal studies. International Journal of Environmental Research and Public Helath. 2019;16(16). doi: 10.3390/ijerph16162884.

20. Gunasekara FI, Carter K, Blakely T. Change in income and change in self-rated health: Systematic review of studies using repeated measures to control for confounding bias. Social Science and Medicine. 2011;72(2):193-201-. doi: 10.1016/j.socscimed.2010.10.029. PubMed PMID: edselc.2-52.0-78650919342.

21. Prus SG. Comparing social determinants of self-rated health across the United States and Canada. Social Science & Medicine. 2011;73(1):50-9. doi: https://doi.org/10.1016/j.socscimed.2011.04.010.

22. Fleming PJ, Spolum MM, Lopez WD, Galea S. The Public Health Funding Paradox: How funding the problem and solution impedes public health progress. Public Health Reports.

2021;136(1):10-3. Epub 20201111. doi: 10.1177/0033354920969172. PubMed PMID: 33176109; PubMed Central PMCID: PMCPMC7856371.

23. Bernet PM. The increasing importance of public health funding research. Journal of Public Health Management and Practice. 2012;18(4):303-5. doi: 10.1097/PHH.0b013e31825aadc5.

24. Mays GP, Smith SA. Evidence links increases in public health spending to declines in preventable deaths. Health Aff (Millwood). 2011;30(8):1585-93. Epub 2011/07/23. doi: 10.1377/hlthaff.2011.0196.

25. Gallet CA. The Impact of Public Health Spending on California STD Rates. International Advances in Economic Research. 2017;23(2):149-59. doi: 10.1007/s11294-017-9631-2.

26. Centers for Disease Control and Prevention. The Behavioral Risk Factor Surveillance System (BRFSS)-Overview: BRFSS 2018. 2019.

27. Center for Disease Control and Prevention. Behavioral Risk Factor Surveillance System 2023 [cited 2023 December 12]. Available from: https://www.cdc.gov/brfss/index.html.

28. SHADAC. State Health Compare. In: SHADAC, editor. University of Minnesota2018.

29. Trust for America's Health. Ready or Not: Protecting the public's health from diseases, disasters, and bioterrorism. 2019.

30. Hennessy CH, Moriarty DG, Zack MM, Scherr PA, Brackbill R. Measuring health-related quality of life for public health surveillance. Public Health Reports. 1994;109(5):665-72. PubMed PMID: 7938388; PubMed Central PMCID: PMCPMC1403555.

31. Mazurek K, Ciesla J, Akakpo R. Disparities in health related quality of life among Illinoisans diagnosed with depressive disorder: findings from the 2017 BRFSS. BMC Public Health. 2020;20(1):936. doi: 10.1186/s12889-020-09041-5.

32. Koelker M, Alkhatib K, Briggs L, Labban M, Meyer CP, Dieli-Conwright CM, et al. Impact of exercise on physical health status in bladder cancer patients. Canadian Urological Association Journal. 2023;17(1):E8-e14. doi: 10.5489/cuaj.8008.

33. United States Census Bureau. American Community Survey 2018 2023 [cited 2023 December 12]. Available from: https://www.census.gov/programs-surveys/acs/data.html.

34. Meit M, Sellers K, Kronstadt J, Lawhorn N, Brown A, Liss-Levinson R, et al. Governance typology: a consensus classification of state-local health department relationships. Journal of Public Health Management and Practice. 2012;18(6):520-8. doi: 10.1097/PHH.0b013e31825ce90b.

35. Singh SR. Public health spending and population health: A systematic review. American Journal of Preventive Medicine. 2014;47(5):634-40. doi: https://doi.org/10.1016/j.amepre.2014.05.017.

36. Joseph KT, Rice K, Li C. Integrating equity in a public health funding strategy. Journal of Public Health Management and Practice. 2016;22 Suppl 1(Suppl 1):S68-76. doi: 10.1097/phh.0000000000000346.

37. Meyer AM, Davis M, Mays GP. Defining organizational capacity for public health services and systems research. Journal of Public Health Management and Practice. 2012;18(6):535-44. doi: 10.1097/PHH.0b013e31825ce928.

38. Mukherjee K, Santerre RE, Zhang NJ. Explaining the efficiency of local health departments in the U.S.: an exploratory analysis. Health Care Management Science. 2010;13(4):378-87. Epub 20100923. doi: 10.1007/s10729-010-9136-5.

39. Frohlich KL, Potvin L. Transcending the known in public health practice: the inequality paradox: the population approach and vulnerable populations. American Journal of Public Health. 2008;98(2):216-21. Epub 2008/01/04. doi: 10.2105/ajph.2007.114777.

40. Link BG, Phelan J. Social conditions as fundamental causes of disease. Journal of Health and Social Behavior. 1995:80-94. doi: 10.2307/2626958.

Figure

Click here to access/download;Figure;Fig1.tif

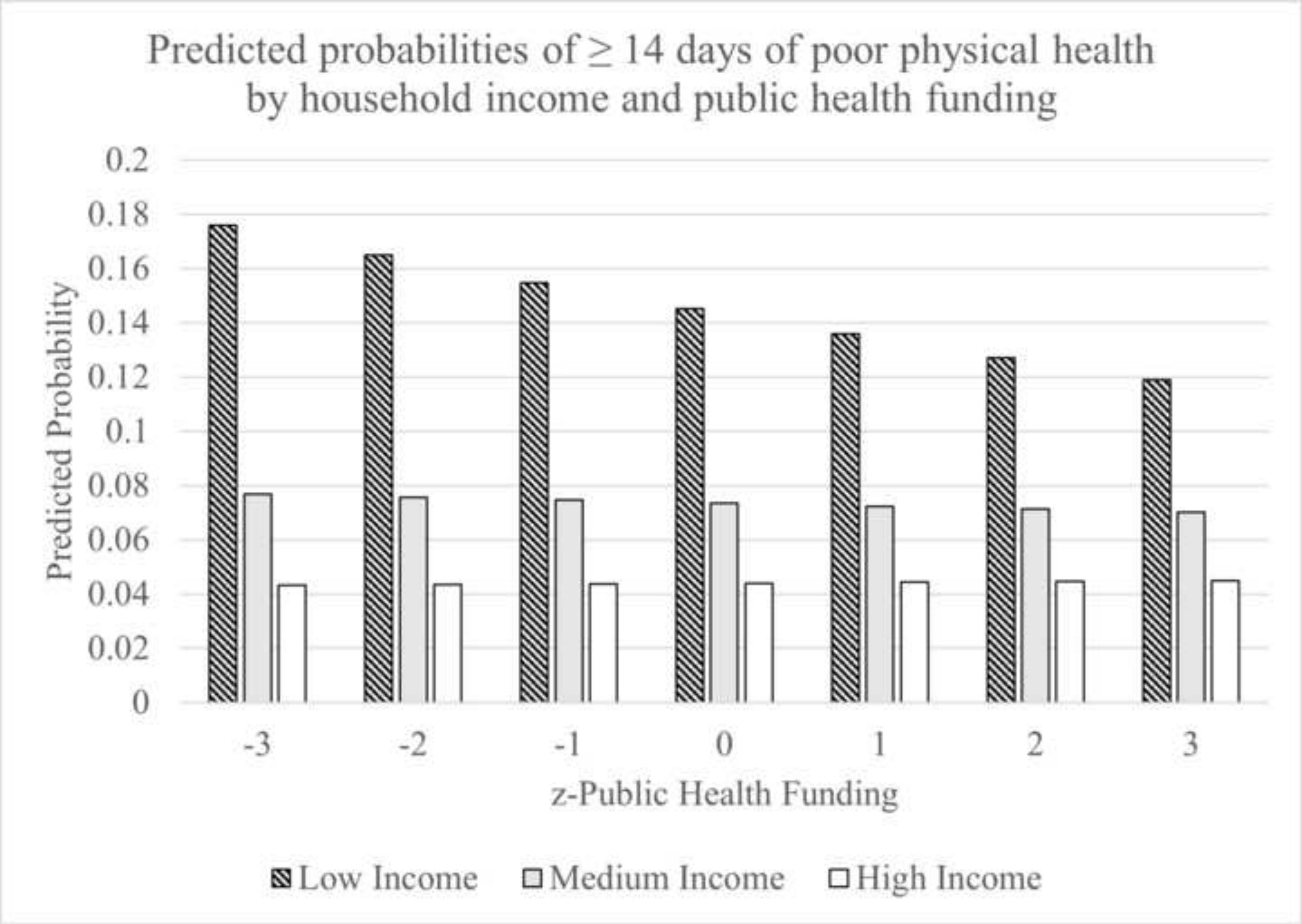

Click here to access/download;Figure;Fig2.tif

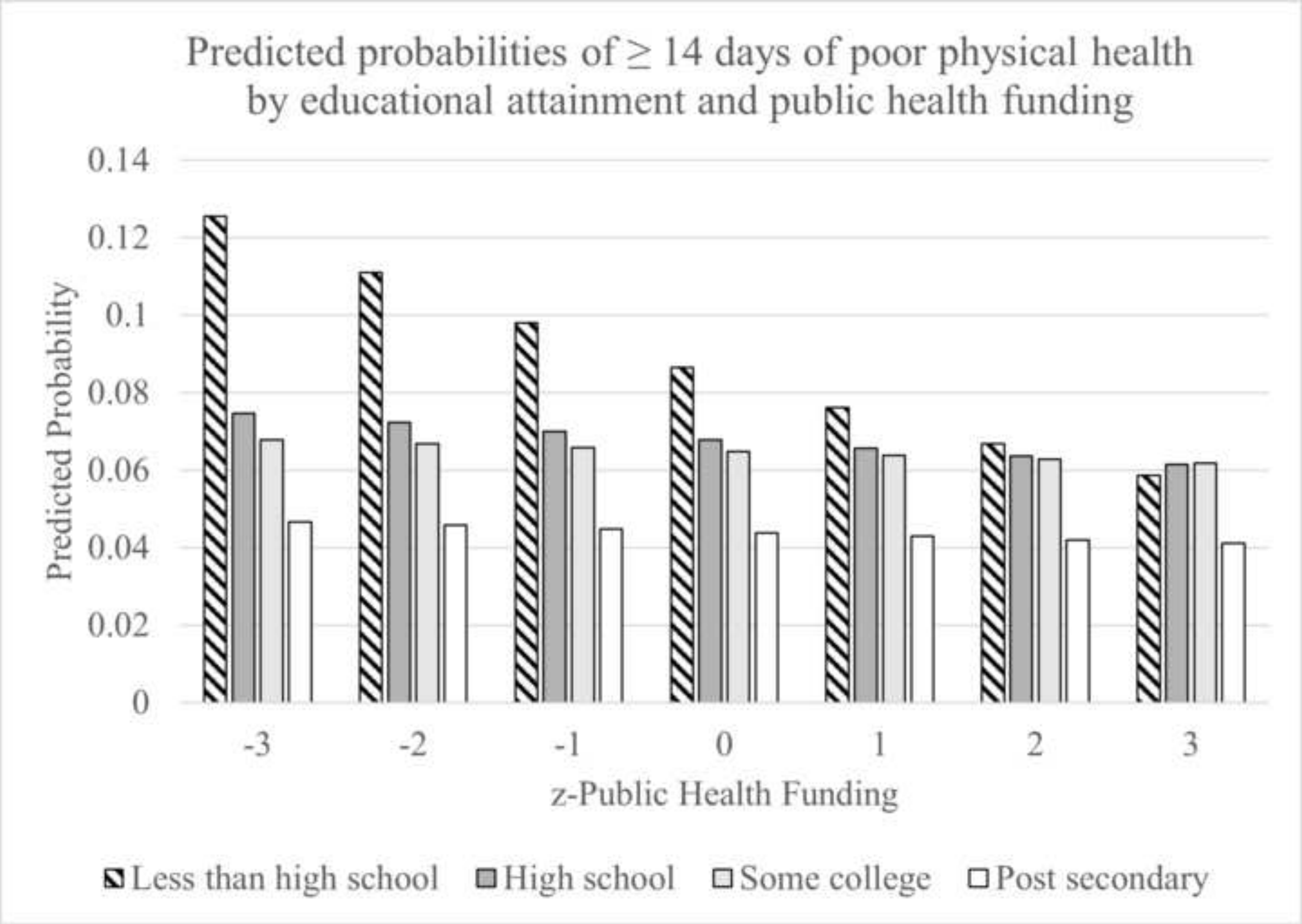

Click here to access/download
Other
Ethics letter.pdf
