# Supplementary material for: The cross-sectional association between state-level public health funding per capita and physical health among adults in the United States
Source: PLoS One. 2025 Apr 1;20(4):e0320920. doi: 10.1371/journal.pone.0320920 (PMC11960902; doi:10.1371/journal.pone.0320920)
Supplement: Attachment — Submitted filename: Response to Academic Editor.docx [file pone.0320920.s004.docx]

We would like to thank the Academic Editor for providing us with a chance to revise our manuscript. We found the Academic Editor’s guidance helpful and have incorporated their suggested changes in the Introduction and Methods sections of the manuscript. We have also revised the entire manuscript for clarity and feel the readability has been improved.
